# Identification and Dissection of a Complex DNA Repair Sensitivity Phenotype in Baker's Yeast

**DOI:** 10.1371/journal.pgen.1000123

**Published:** 2008-07-11

**Authors:** Ann Demogines, Erin Smith, Leonid Kruglyak, Eric Alani

**Affiliations:** 1 Department of Molecular Biology and Genetics, Cornell University, Ithaca, New York, United States of America; 2 Lewis-Sigler Institute for Integrative Genomics, Princeton University, Princeton, New Jersey, United States of America; 3 Department of Ecology and Evolutionary Biology, Princeton University, Princeton, New Jersey, United States of America; The Jackson Laboratory, United States of America

## Abstract

Complex traits typically involve the contribution of multiple gene variants. In this study, we took advantage of a high-density genotyping analysis of the BY (S288c) and RM strains of *Saccharomyces cerevisiae* and of 123 derived spore progeny to identify the genetic loci that underlie a complex DNA repair sensitivity phenotype. This was accomplished by screening hybrid yeast progeny for sensitivity to a variety of DNA damaging agents. Both the BY and RM strains are resistant to the ultraviolet light–mimetic agent 4-nitroquinoline 1-oxide (4-NQO); however, hybrid progeny from a BY×RM cross displayed varying sensitivities to the drug. We mapped a major quantitative trait locus (QTL), *RAD5*, and identified the exact polymorphism within this locus responsible for 4-NQO sensitivity. By using a backcrossing strategy along with array-assisted bulk segregant analysis, we identified one other locus, *MKT1*, and a QTL on Chromosome VII that also link to the hybrid 4-NQO–sensitive phenotype but confer more minor effects. This work suggests an additive model for sensitivity to 4-NQO and provides a strategy for mapping both major and minor QTL that confer background-specific phenotypes. It also provides tools for understanding the effect of genetic background on sensitivity to genotoxic agents.

## Author Summary

Complex traits often display a range of phenotypes due to the contribution of multiple gene variants. Advances in statistical models, genetic mapping, and DNA genotyping and sequencing have made baker's yeast an excellent system to identify quantitative trait loci (QTL), regions of the genome linked to a quantitative phenotypic trait. We focused on a complex DNA damage sensitivity phenotype in yeast in which parental strains are unaffected but give rise to progeny with a sensitive phenotype. We used a whole-genome approach to isolate defects in DNA repair caused by gene variants in two strains of baker's yeast that display approximately 0.5% sequence divergence. The parental strains are resistant to the ultraviolet light–mimetic agent 4-nitroquinoline 1-oxide (4-NQO); however, a large number of spore progeny displayed varying sensitivities to the drug. Through linkage and bulk segregant analyses we identified one major QTL, *RAD5*, and two minor QTL linked to sensitivity to 4-NQO, and we provide evidence that sensitivity is due to additive effects involving several QTL. These observations provide a powerful model in which to understand the basis of disease penetrance and how phenotypic variation can be mapped at the gene level.

## Introduction

Complex traits often display phenotypic variation due to additive and interactive effects of gene variants located at multiple loci. Because of the above complications, only a relatively small number of quantitative trait loci (QTL) have been identified [1]–[7]. Baker's yeast has become an excellent system to model and dissect complex traits. For example, Steinmetz et al. [8], Brem et al. [9], and Perlstein et al. [10] took advantage of the natural variation between wild and laboratory yeasts to dissect the complex phenotypes that underlie high-temperature growth, transcriptional regulation, and drug sensitivity, respectively. Steinmetz et al. [8] mapped a trait present in one parental strain while Brem et al. [9] mapped transcriptional traits in both parents and hybrid progeny by looking for linkage between phenotypic variation and specific molecular markers. Perlstein et al. [10] performed linkage analysis to identify QTL linked to sensitivity to small molecule drugs. Such analyses involved the use of high-density oligonucleotide arrays to analyze a large collection of F2 hybrid progeny.

Complex traits can be identified in one parental background and mapped by crossing the strain to one lacking the phenotype. However, a complex phenotype or disease can be caused by variants of genes derived from different strain backgrounds, resulting in offspring showing phenotypic variation not present in the parents. Such complex phenotypes have been shown to affect the penetrance of human cancers (e.g. [11]–[13]). For example, in rare cases of hereditary non-polyposis colorectal cancer, the disease is thought to result from the combined effect of non-pathogenic DNA mismatch repair alleles [13]. Such phenotypes could be due to impaired protein/protein interactions or other molecular interactions that occur only in the hybrid progeny; alternatively, they could be due to additive effects involving several QTL.

The above observations encouraged us to identify and dissect a complex trait in baker's yeast associated with defects in DNA repair. We focused on such phenotypes because of our interest [14] in understanding how DNA damage repair pathways contribute to maintaining genomic stability (reviewed in [15],[16]). Also, identifying loci that underlie such traits provides a model to explain differences in the penetrance of phenotypes observed in humans (e.g. [3],[8],[17]). We used a whole-genome approach to identify such loci in the meiotic progeny of a cross between the BY (S288c) and RM strains of *S. cerevisiae*. BY is a commonly used lab strain and RM is a wild isolate from a grape vineyard in California that displays approximately 0.5–1% sequence divergence relative to S288c [9],[18]. We tested RM and BY strains and the meiotic progeny of a BY/RM diploid for sensitivity to a variety of DNA damaging agents. Both the BY and RM strains are resistant to the ultraviolet light-mimetic agent 4-nitroquinoline 1-oxide (4-NQO); however, a large number of spore progeny from the BY/RM diploid showed varying sensitivity to the drug. Through linkage analysis and a backcrossing strategy involving a bulk segregant analysis and SNP genotyping, we identified one major and two minor QTL linked to the 4-NQO-sensitive phenotype. These observations provide a powerful model in which to understand the basis of disease penetrance and how genetic variation can be mapped at the gene level.

## Results

### Complex Molecular Interactions Are Observed in BY/RM Hybrid Progeny Treated with DNA Damaging Agents

To identify novel complex traits in *Saccharomyces cerevisiae* associated with DNA repair, we examined 123 haploid meiotic progeny of a BY/RM diploid [9] for sensitivity to DNA damaging agents (Tables 1, 2; Materials and Methods). Using high-density oligonucleotide arrays, 2956 genetic markers were identified between the two parental strains that cover over 99% of the genome [9],[19]. Meiotic spore progeny from the BY/RM hybrid were genotyped using the same high-density oligonucleotide array, creating an inheritance map for each of the 123 hybrid spore progeny [9],[20],[21].

**Table 1:** Phenotypes of BY/RM hybrid progeny.

			Sensitivity Phenotype		
Segregant		MMS	4-NQO	Bleomycin	Caffeine
	BY	+	+	+	+
	RM	--	+	+	+
1	1-1-d	+	++	±	-
2	1-2-d	-	++	+	±
3	1-3-d	-	-	++	+
4	1-4-d	+	++	-	-
5	1-5-c	-	-	-	+
6	2-1-d	+	++	-	±
7	2-2-d	--	-	++	+
8	2-3-d	+	++	±	+
9	2-4-a	-	+	±	±
10	2-5-d	--	-	++	+
11	2-6-d	+	±	±	±
12	2-7-d	--	±	±	++
13	3-1-d	--	±	-	-
14	3-2-d	±	±	-	-
15	3-3-d	±	±	++	+
16	3-4-d	-	-	±	±
17	3-5-d	--	-	+	-
18	4-1-c	--	±	--	+
19	4-2-a	+	+	±	-
20	4-3-d	--	-	+	+
21	4-4-d	++	++	+	+
22	5-1-d	±	++	-	±
23	5-2-d	--	-	+	+
24	5-3-d	--	±	+	+
25	5-4-d	--	±	+	±
26	5-5-d	+	+	+	+
27	6-1-d	±	-	+	±
28	6-2-b	±	+	+	±
29	6-3-c	--	±	±	±
30	6-4-d	+	++	++	+
31	6-5-d	-	±	+	±
32	6-6-d	-	±	-	-
33	6-7-d	+	+	-	±
34	7-1-d	+	++	±	-
35	7-2-c	--	±	+	+
36	7-3-d	+	++	+	+
37	7-4-c	++	++	±	++
38	7-5-d	-	+	+	+
39	7-6-c	--	±	+	+
40	7-7-c	--	±	+	+
41	7-8-d	--	±	+	±
42	8-1-a	-	+	++	++
43	8-2-d	+	++	-	-
44	8-3-a	++	+	-	+
45	8-4-c	--	++	+	±
46	8-5-b	++	++	±	+
47	8-6-c	--	--	-	+
48	8-7-b	±	+	-	±
49	9-1-d	+	++	±	±
50	9-2-d	--	±	±	-
51	9-3-d	--	±	++	+
52	9-4-d	-	++	+	+
53	9-5-d	--	-	±	±
54	9-6-d	--	--	±	+
55	9-7-d	--	±	±	±
56	10-1-c	+	++	-	-
57	10-2-d	-	++	+	+
58	10-3-c	-	++	±	-
59	10-4-d	+	++	-	±
60	11-1-a	+	+	±	+
61	11-2-d	-	-	±	+
62	11-3-b	-	+	±	-
63	12-1-d	-	-	-	±
64	12-2-b	++	++	-	+
65	13-1-a	--	-	+	±
66	13-2-c	--	-	-	-
67	13-3-b	+	++	±	±
68	13-4-a	±	±	-	+
69	13-5-c	+	++	±	-
70	14-1-b	--	-	-	±
71	14-2-c	±	+	±	+
72	14-3-d	±	+	+	±
73	14-4-a	--	±	-	-
74	14-5-b	+	++	±	±
75	14-6-d	-	++	±	-
76	14-7-c	--	-	±	±
77	15-2-d	+	±	-	+
78	15-3-b	-	±	+	±
79	15-4-d	+	+	-	+
80	15-5-b	--	-	--	±
81	15-6-c	±	++	--	-
82	16-1-d	-	±	±	±
83	17-1-a	+	±	-	±
84	17-2-d	--	-	+	+
85	17-4-a	--	-	±	±
86	17-5-b	+	+	+	+
87	18-1-d	+	++	--	--
88	18-2-d	±	-	±	+
89	18-3-d	--	-	--	-
90	18-4-c	±	+	±	±
91	18-6-d	+	++	-	+
92	19-1-c	--	-	++	+
93	19-2-c	--	-	++	±
94	19-3-c	++	+	++	++
95	19-4-b	+	+	-	+
96	19-5-b	+	++	+	-
97	20-1-d	+	+	+	-
98	20-2-d	+	+	++	-
99	20-3-c	--	-	±	+
100	20-4-c	--	++	++	+
101	20-5-d	-	±	++	+
102	21-1-d	+	-	-	+
103	21-2-d	+	++	--	±
104	21-3-d	--	-	-	+
105	21-4-d	±	±	±	+
106	21-5-c	nt	nt	++	nt
107	22-1-d	--	±	-	+
108	22-2-d	++	++	++	±
109	22-3-b	++	+	±	±
110	22-4-d	+	±	+	±
111	22-5-d	+	+	++	++
112	23-2-d	+	+	-	±
113	23-3-d	±	+	-	+
114	23-4-d	--	-	-	±
115	23-5-d	--	±	+	+
116	24-1-d	--	-	-	-
117	25-1-d	--	-	-	±
118	25-3-d	--	-	-	±
119	25-4-d	--	±	±	±
120	25-5-d	+	+	++	±
121	26-1-d	+	+	-	±
122	26-2-d	±	±	±	+
123	26-3-d	+	±	-	-

RM, BY and RM/BY meiotic spore progeny were tested in triplicate using a plating assay and scored for resistance to the DNA damaging agents, MMS, 4-NQO, bleomycin and caffeine (See Materials and Methods). Strains were scored as follows: very sensitive (- -), sensitive (-), slightly sensitive (±), wild-type resistance (+) and increased resistance (+ +). nt, not tested.

**Table 2:** Strains.

Strain	Background	Relevant Genotype
BY4716	BY	*MATα, lys2Δ*
RM11-1a	RM	*MATa, leu2Δ, ura3Δ, ho::Kan*
EAY253	S288c	*MATα ura3-52, leuΔΔ1, his3Δ200, rad52Δ::LEU2*
EAY1463	BY	*MATα, lys2Δ, RAD5-RM::NatMX*
EAY1465	BY	*MATα, lys2Δ, RAD5-BY::NatMX*
EAY1467	RM	*MATa, leu2Δ, ura3Δ, ho::Kan, RAD5-BY::NatMX*
EAY1469	RM	*MATa, leu2Δ, ura3Δ, ho::Kan, RAD5-RM::NatMX*
EAY1471	BY	*MATα, lys2Δ, RAD5-I791S::KanMX*
EAY2169	BY	*MATα, lys2Δ, RAD5-E783D::KanMX*
EAY2295	3D-BK3-12	*MATa, lys2Δ, ura3Δ, leu2Δ, RAD5-RM*
EAY2298	3D-BK3-52	*MATα, lys2Δ, ura3Δ, RAD5-RM*
EAY2336	3D-BK3-16	*MATa, lys2Δ, ura3Δ, leu2Δ, RAD5-RM*
EAY2317	3D-BK3-12	*MATa, lys2Δ, ura3Δ, leu2Δ, RAD5-RM, mkt1Δ::NatMX*
EAY2323	3D-BK3-52	*MATα, lys2Δ, ura3Δ, RAD5-RM, mkt1Δ::NatMX*

**Figure 1:**
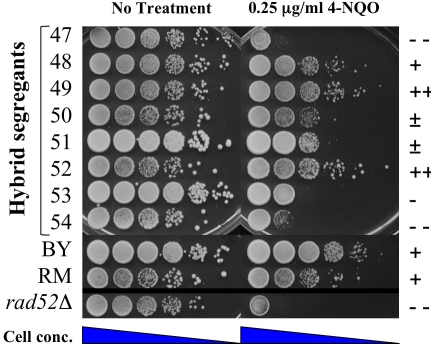
BY/RM hybrid progeny show altered sensitivity to the DNA damaging agent, 4-NQO. Phenotype testing of BY-RM hybrid segregants. Saturated cultures of BY, RM, *rad52Δ* (4-NQO control), and representative progeny of the BY×RM cross (segregants 47–54) were diluted in water and spotted in 10-fold serial dilution (undiluted to 10^−5^) onto YPD with or without 0.25 µg/ml 4-NQO. Plates were photographed after 2-day incubation at 30 C. Strains were scored as very sensitive (- -), sensitive (-), slightly sensitive (±), wild-type resistance (+) and increased resistance (+ +). The scoring method was also used in Table 1 and Figures 3, 4, 6, and 7.

We looked for a reproducible DNA damage sensitivity phenotype in the hybrid progeny that was not seen in either parental strain. This phenotype was assessed by plating serial dilutions of saturated cultures from each hybrid segregant onto plates containing varying concentrations of DNA damaging agents (Table 1 and Figure 1). The DNA damaging agents tested included methyl methane sulfonate (MMS), a DNA alkylating agent, 4-nitroquinoline 1-oxide (4-NQO), an ultraviolet light (UV) mimetic, bleomycin, a radiomimetic antitumor drug, and caffeine, a compound that sensitizes cells to genotoxic agents [22]. We identified hybrid progeny that displayed varying sensitivities to 4-NQO, bleomycin, and caffeine treatments (Table 1). Surprisingly, the RM parent displayed sensitivity to MMS, and this sensitivity was observed in half of the progeny (Table 1); however, as shown below this phenotype does not appear to be entirely monogenic.

### Linkage Analysis of DNA Damage Phenotypes

**Figure 2:**
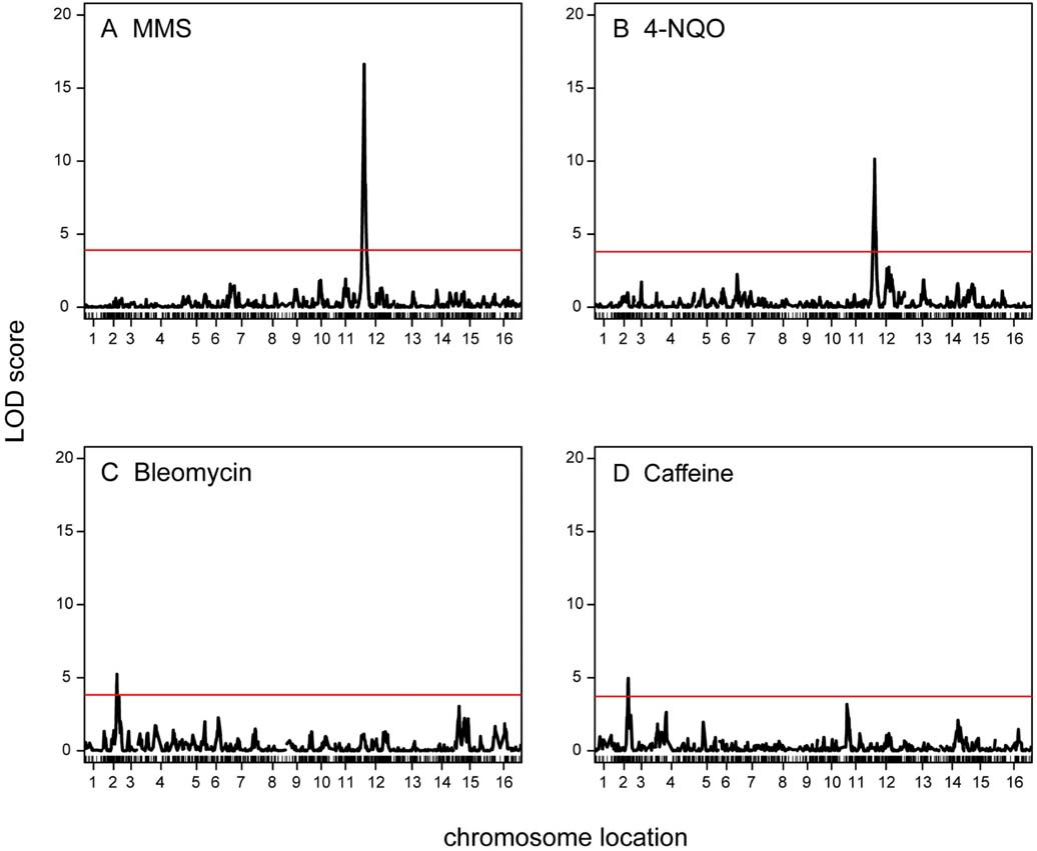
Linkage analysis shows that the MMS- and 4-NQO-sensitive phenotypes observed in hybrid progeny are linked to a locus on chromosome 11. Linkage analysis for A-D was performed using R/qtl. Linkage analysis for sensitivity to MMS (A), 4-NQO (B), bleomycin (C) and caffeine (D) are presented. The red line in each panel indicates a significance cutoff where p = 0.0125 (0.05/the number of traits: 4, Materials and Methods).

We performed linkage analysis for each of the phenotypes tested (Figure 2), determined significance cutoffs for each trait by permutation, and calculated support intervals (see Materials and Methods). MMS-sensitivity, which was seen in the RM parent and half of the hybrid segregants tested, showed strong linkage to a chromosome 12 locus near *SMF3* (Figure 2A, LOD score of 16.6 for YLR034C_1989). Linkage analysis of 4-NQO-sensitivity identified the same region (LOD score 10.1 for YLR034C_1989, support intervals completely overlap), but in this case the phenotype was seen in the hybrid progeny but not in the parental strains, indicating that 4-NQO-sensitivity involves additional loci (Figure 1B, Table 1). The bleomycin-sensitive phenotype showed linkage to chromosome 2 (LOD score 5.2 for YBR138C_275; Figure 2C). The caffeine-sensitive phenotype also showed linkage to chromosome 2 (LOD score 5.0 for YBR161W_293; Figure 2D). Both loci overlap a region previously linked to growth-related transcripts and daughter cell separation [19]; however, the linkages described here are not likely to be due to the same locus because their support intervals do not overlap. We further pursued the MMS- and 4-NQO-sensitivity linkages because they showed the highest statistical significance.

### A Single Polymorphism in *RAD5* Confers Sensitivity to MMS and Contributes to the hybrid 4-NQO Sensitivity Phenotype

Twenty-two genes lie within the support interval associated with the MMS-sensitive and 4-NQO sensitive phenotypes. A prime candidate is *RAD5*, located ∼2 kb away from the peak marker. *RAD5* encodes a DNA-dependent ATPase involved in the error-free branch of post-replicative repair [23]. *rad5* null mutants are sensitive to MMS, UV, and ionizing radiation [24],[25]. Only one other gene within the region is required for resistance to MMS, *AAT2*, which encodes an aspartate aminotransferase involved in nitrogen metabolism [25],[26]. It is located on the edge of the region, approximately 12.5 kb from the peak marker. Although required for MMS-resistance, *aat2* deletion strains are not UV-sensitive [25].

**Figure 3:**
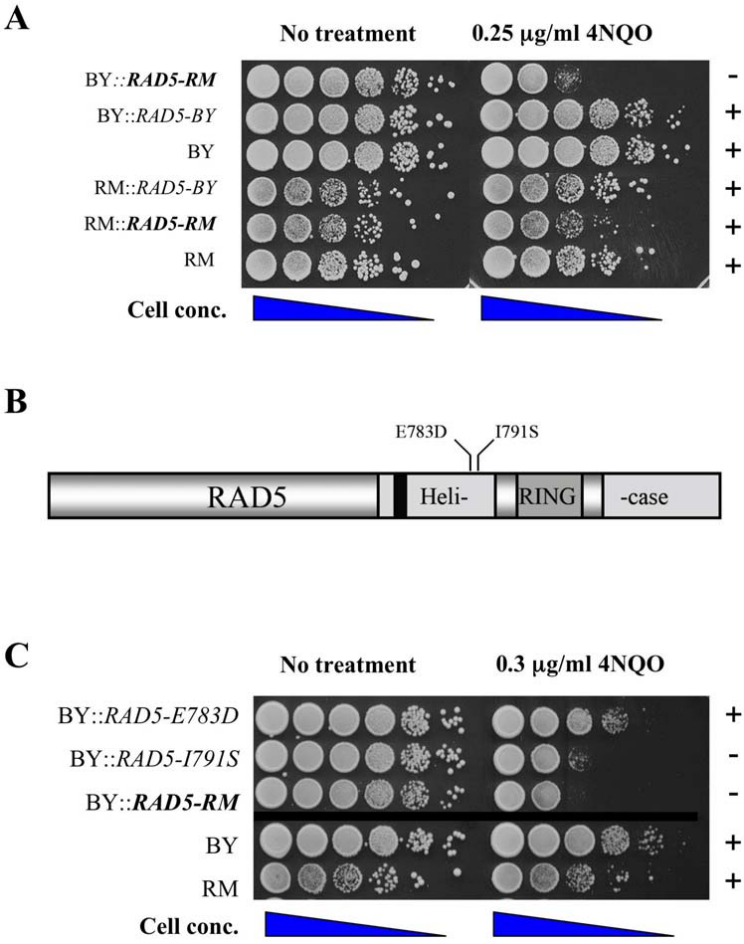
Homologous replacement of *RAD5* in the BY background recreates the 4-NQO-sensitive phenotype. A Saturated cultures of BY, RM, and parental strains containing *RAD5-(BY* or *RM)::NatMX* were diluted in water and spotted in 10-fold serial dilution (undiluted to 10^−5^) onto YPD with or without 0.25 µg/ml 4-NQO. Plates were photographed after 2-day incubation at 30 C. B RAD5 polymorphisms in the BY and RM strains. The first letter for each polymorphism indicates the BY polymorphisms (E783, I791) and the second RM (D783, S791). C Single RAD5 polymorphisms tested in the BY strain background for 4-NQO sensitivity. The assay described in Panel A was used.

Based on the above information, we focused on *RAD5* as a candidate gene associated with MMS and 4-NQO sensitivity observed in the hybrid progeny. We tested the role of *RAD5* polymorphism by homologous allele replacement and plasmid suppression (Figures S1and S2) approaches. RM strains, which are sensitive to MMS, became resistant when the *RAD5* gene in RM was replaced with the BY copy. In addition, the MMS-sensitivity observed in RM strains could be created in the BY strain by replacing *RAD5* with the RM copy (Figure S1). Replacement of the BY open reading frame of *RAD5* with the *RM* allele also re-created the 4-NQO sensitive phenotype in the BY strain (BY*::RAD5-RM;* Figure 3A, Materials and Methods). The sensitivity was not observed when the *RAD5* gene in the RM strain was replaced with the BY copy (RM*::RAD5-BY*), consistent with the RM copy of *RAD5* being associated with 4-NQO sensitivity. These data indicate that that the RM copy of *RAD5* contains polymorphisms that confer the MMS-sensitive phenotype and contribute to sensitivity to 4-NQO. Interestingly, although the MMS-sensitivity phenotype appeared to be monogenic and showed strong linkage to *RAD5*, the locus does not completely account for the phenotype. Genotyping analysis of the 121 meiotic progeny indicated that although there is a very strong linkage to *RAD5*, there is overlap in which progeny displaying moderate (−, −/+) sensitivity to MMS fell into both the *RAD5-BY* and *RAD5-RM* genotype classes (data not shown).

The *RAD5* open reading frames in BY and RM differ by two non-synonymous substitutions that map to amino acid positions 783 (glutamic acid in BY, aspartic acid in RM) and 791 (isoleucine in BY, serine in RM). These polymorphisms both map to the helicase domain of RAD5 (Figure 3B; [23]). Using site-directed mutagenesis and homologous gene replacement methodologies we found that the serine residue at position 791 in RM RAD5 contributed to sensitivity to 4-NQO. This was accomplished by introducing the *RAD5-I791S* substitution into the BY strain and showing that the resulting strain was sensitive to 4-NQO (Figure 3C, Materials and Methods). This allele was also responsible for the MMS-sensitivity phenotype observed in the RM parental strain (Figure S3). No other strains of *Saccharomyces cerevisiae* that have been sequenced (http://www.sanger.ac.uk/Teams/Team71/durbin/sgrp/) contain this serine 791 polymorphism. We also phenotype-tested a number of wild yeast strains in our collection and none of them showed sensitivity to MMS equivalent to that observed in the RM parental strain (data not shown). Finally, this polymorphism is not the same as the loss of function mutation that was previously identified in the W303-derived copy of *RAD5*, G535R [27].

### No Candidate Genes Show Suppression of the Hybrid 4-NQO-Sensitive Phenotype

We identified one locus, *RAD5*, from the RM strain involved in sensitivity to 4-NQO. The fact that the parental strains were resistant to 4-NQO indicated that at least one locus from BY was also required. We searched for additional loci by partitioning the segregants by their genotype at *RAD5* and performed linkage analysis on the subgroups, but there were no regions showing a significant linkage. This negative result could simply reflect the reduced sample size of each subgroup.

A candidate gene approach was also pursued to identify additional factors from the BY parent involved in the sensitivity phenotype. Because we were able to recreate the 4-NQO hybrid sensitivity phenotype by replacing *RAD5* in the BY strain with the RM copy, we used the BY::*RAD5-RM* strain to search for candidate RM genes that could suppress the 4-NQO-sensitive phenotype. We pursued this approach because we were able to suppress the 4-NQO-sensitive phenotype of the *BY::RAD5-RM* strain by introducing a single-copy *ARS CEN* plasmid containing *RAD5-BY* (data not shown). Single-copy plasmids containing the entire open reading frame and ∼250–500 bp of downstream and upstream sequence from the RM parent strain were created and transformed into the *BY::RAD5-RM* strain (Materials and Methods). Plasmids were made containing several candidate genes that were shown to physically and/or genetically interact with *RAD5*. These include *POL30* and *RAD18*, whose gene products interact with RAD5 through two-hybrid analysis [28],[29], and *CTF18*, *CSM3*, *RAD50* and *TOF1*, mutations in which are synthetically lethal with *rad5* mutations [30]. None of these candidate genes derived from the RM parent strain suppressed the 4-NQO-sensitivity of the *BY::RAD5-RM* strain (Figure S4; data not shown).

### 4-NQO-Sensitive Phenotype Is Maintained after Several Backcrosses

**Figure 4:**
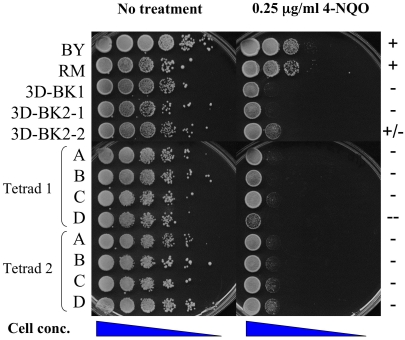
Phenotypic variation is seen in backcrossed 4-NQO-sensitive segregants mated to each other. Two 4-NQO-sensitive spore clones (3D-BK2-1, 3D-BK2-2) from the first backcross (3D-BK1×RM) were mated and the resulting progeny were tested for sensitivity to 4-NQO. The progeny are shown as Tetrads 1 and 2. Saturated cultures of these progeny were diluted in water and spotted in 10-fold serial dilution (undiluted to 10^−5^) onto YPD with or without 0.25 µg/ml 4-NQO. Plates were photographed after 2-day incubation at 30 C. BY and RM strains were tested as controls.

A backcrossing strategy was pursued to identify a subset of BY loci linked to 4-NQO-sensitivity. To isolate these loci we backcrossed the BY*::RAD5*-RM strain to the RM parent. Because both parental strains contained the *RAD5-RM* allele, all progeny from this cross have the potential to show sensitivity. BY*::RAD5-*RM was crossed to the RM parental strain and a sensitive spore from this cross was backcrossed to the RM parent (3D-BK1, Figure 4). This backcross was repeated two more times (progeny labeled BK2 and BK3 for second and third backcross, respectively). After each backcross, haploid hybrid progeny were phenotype tested and segregants displaying sensitivity to 4-NQO were chosen and backcrossed to RM. After the first backcross, roughly 35% of the progeny displayed the 4-NQO-sensitive phenotype. Even after the third backcross a 2:2 4-NQO sensitive: resistant segregation pattern for each tetrad was not seen, suggesting that a large number of loci modify sensitivity to 4-NQO. At the end of backcrossing, the parental contribution of the final segregants was approximately 94% RM and 6% BY.

To determine if multiple genetic loci were segregating in the second backcross, two 4-NQO-sensitive segregants from the second backcross (∼12.5% BY and ∼87.5% RM) were mated and sporulated. Incomplete complementation (varying degrees of sensitivity) of the 4-NQO-sensitive phenotype was seen in progeny from this cross and in progeny from other crosses involving second backcross segregants (Figure 4; data not shown). Thus, multiple loci that contribute to the hybrid 4-NQO-sensitive phenotype are likely to be segregating in the second backcross because all the progeny from this cross display some degree of sensitivity (Figure 4).

### Microarray-Assisted Bulk Segregant Analysis Implicates Two Genomic Loci in the 4-NQO Phenotype

**Figure 5:**
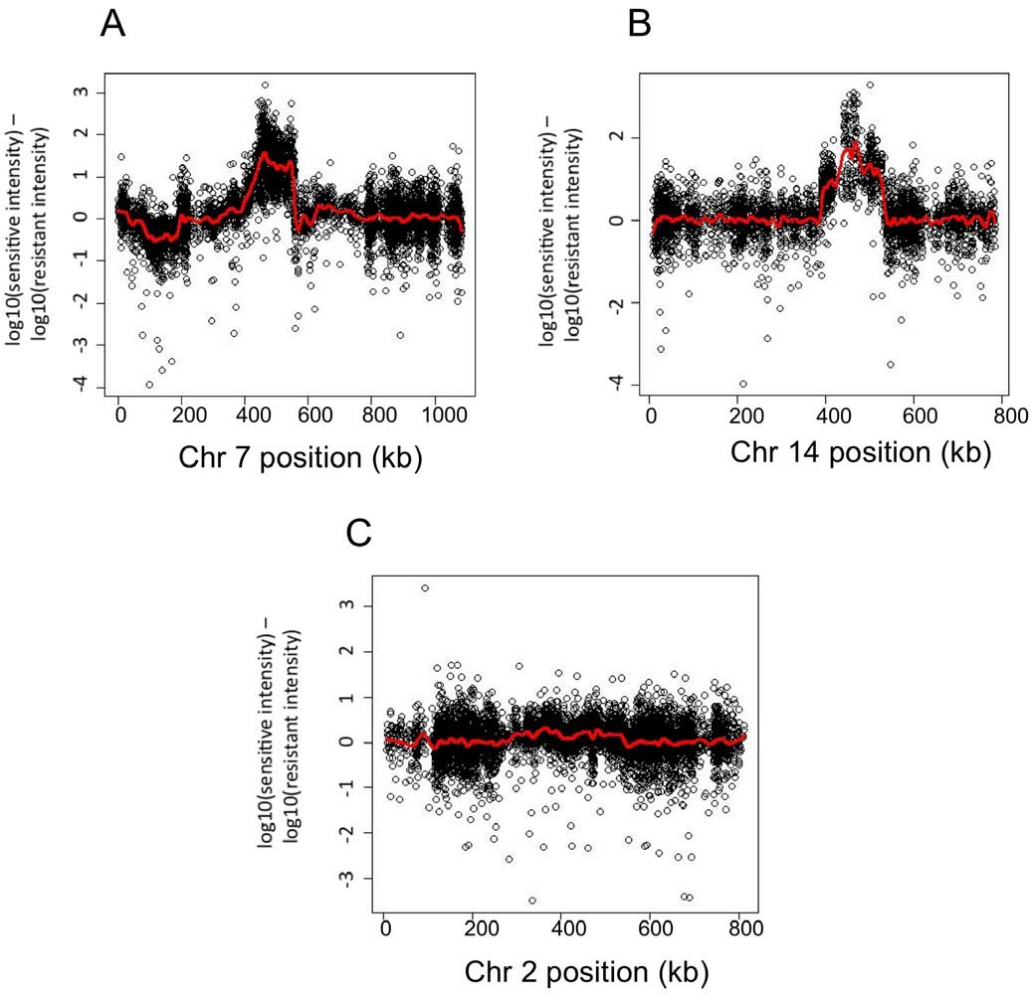
Two regions are associated with the 4-NQO-sensitive phenotype. DNA from pools of forty backcrossed individuals (BK3) either resistant or sensitive to 4-NQO was hybridized to Affymetrix tiling arrays. The difference in intensity values was calculated for every probe that was considered to contain a sequence variant between BY and RM by SNPscanner [51]. The log_10_ (intensity difference) was plotted along the chromosome and smoothed. Chromosomes that displayed regions where the smoothed value exceeded +/−1 are shown in panels A and B. Panel C is a representative image of the chromosomes that did not show linkage regions.

Bulk segregant analysis followed by microarray SNP genotyping was used to identify loci that conferred 4-NQO sensitivity in the third backcross lines. Genomic DNA was isolated from pooled resistant and sensitive segregants (40 from each set). The two pools were then hybridized to an Affymatrix reference sequence microarray that allows for the determination of inheritance between the two parental strains. Mapping was then performed (Materials and Methods; [31],[32]). If a locus is unlinked to the phenotype, it will not show preferential inheritance in either pooled sample and therefore will show an averaged baseline of inheritance (Figure 5C). For a linked locus, the genomic DNA in one pool will be enriched for one parental inheritance, and therefore will show a peak of inheritance when comparing the array results across the pooled progeny of the phenotype (Figures 5A and B). This method allowed us to screen a large number of segregants and measure the average genotype of progeny within each pool. From this analysis, two regions were identified that segregated preferentially with the 4-NQO phenotype (Figure 5). These regions are on chromosome 7, between 433,546 bp and 551,683 bp (smoothed value of >1), with the peak at 463,019 bp, and on chromosome 14, between 437,935 bp and 473,640 bp (smoothed value of >1.5), with the peak at 467,209 bp (Figure 5A and B). The regions mapped are large, approximately 118 kb and 36 kb in length for chromosome 7 and 14, respectively. This may be due to the backcrossing and the nature of pooled segregant analysis, but also could be due to several genes within each region contributing to the phenotype. We focused on candidate genes located near the peak of each region.

### BY-Derived *MKT1* Contributes to Hybrid 4-NQO-Sensitive Phenotype

For the chromosome 7 linkage region, the peak includes *KAP122*, which encodes a protein involved in importing the TOA1-TOA2 complex into the nucleus. This gene has been implicated in regulation of pleiotropic drug resistance [33], making it an excellent candidate to test for suppression. A second candidate gene, *PDR1*, maps to within 6 kb of the peak and was chosen because it also encodes a protein involved in the pleiotropic drug response [34]. Due to the large size of this region (>100 kb), we focused our analysis on these two genes. For the chromosome 14 linkage region, the peak lies near *MKT1*, which was named for it role in the maintenance of K2 killer toxin [35], and has been implicated in the posttranscriptional regulation of HO endonuclease [36]. This region has also been implicated in high temperature growth, sporulation, and expression quantitative traits [3],[37],[38],[39]. Sequence analysis of the RM and BY strains indicated that *KAP122, PDR1*, and *MKT1* each contain non-synonymous and synonymous polymorphisms within their coding regions.

**Figure 6:**
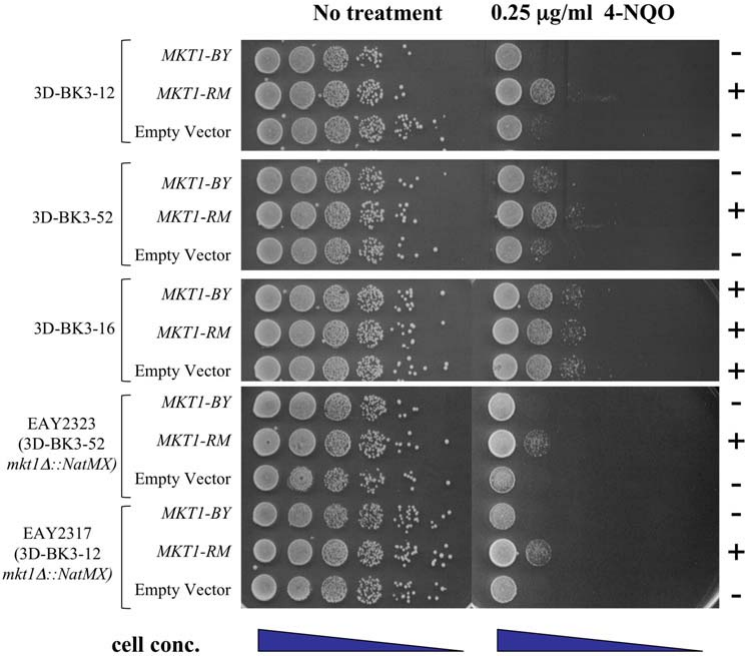
*MKT1* shows allele-specific suppression of 4-NQO-sensitivity in backcrossed segregants. Single-copy *ARS CEN* vectors containing BY or RM derived *MKT1* were introduced into 4-NQO sensitive (3DBK3-12 and 3DBK3-52) and resistant (3DBK3-16) strains from the 3^rd^ backcross. Single-copy *ARS CEN* vectors containing BY or RM derived *MKT1* were also introduced into *mkt1Δ* derivatives of 3DBK3-12 (EAY2317) and 3DBK3-52 (EAY2323). Saturated cultures of these transformants were diluted in water and spotted in 10-fold serial dilution (undiluted to 10^−5^) onto minimal selective yeast media with or without 0.25 µg/ml 4-NQO. Plates were photographed after 2-day incubation at 30 C.

*KAP122, PDR1*, and *MKT1* containing ∼500 bp of upstream and downstream sequence were cloned from both the RM and BY strains and inserted into *ARS CEN* plasmids (Table 3). These plasmids were introduced into 4-NQO-sensitive spores derived from the third backcross of BY::*RAD5-RM*×RM (3DBK3-12 and 3DBK3-52, included in the bulk segregant analysis). None of the *KAP122* and *PDR1* plasmids suppressed the 4-NQO phenotype of these segregants (data not shown). However, *MKT1* derived from the RM parent suppressed the 4-NQO phenotype of these segregants and other segregants from the third backcross (Figure 6; data not shown). Neither of the parental derived alleles of *MKT1* conferred increased sensitivity or resistance when inserted into a backcrossed segregant that did not show the 4-NQO-sensitive phenotype (3D-BK3-16, Figure 6).

**Table 3:** Plasmids.

Plasmid	Genotype
pEAA403	*LYS2, ARSH4, CEN6, aatR1::Cm^R^-ccdB::attR2*
pEAA404	*MKT1-BY, LYS2, ARSH4, CEN6*
pEAA405	*MKT1-RM, LYS2, ARSH4, CEN6*
pEAA406	*KAP122-BY, LYS2, ARSH4, CEN6*
pEAA407	*KAP122-RM, LYS2, ARSH4, CEN6*
pEAA408	*PDR1-BY, LYS2, ARSH4, CEN6*
pEAA409	*PDR1-RM, LYS2, ARSH4, CEN6*
pEAA416	*RAD50-RM, LYS2, ARSH4, CEN6*
pEAA430	*CSM3-RM, LYS2, ARSH4, CEN6*
pEAA431	*RAD18-RM, LYS2, ARSH4, CEN6*
pEAA433	*CTF18-RM, KanMX, ARSH4, CEN6*
pEAA434	*PCNA-RM, KanMX, ARSH4, CEN6*
pEAA435	*TOF1-RM, KanMX, ARSH4, CEN6*
pEAI207	*RAD5-BY::KanMX*
pEAI208	*RAD5-RM::KanMX*
pEAI209	*RAD5-BY::NatMX*
pEAI210	*RAD5-RM::NatMX*
pEAI213	*RAD5-I791S::KanMX*
pEAI214	*RAD5-E783D::KanMX*

pEAA403-431, pEAA433-435, and pEAI207-214 were derived from pRS317 [47], pRS414 [47], and pUC19, respectively.

As a further test, we deleted the *MKT1* gene present in the third backcross of *BY::RAD5-RM*×RM (3DBK3-12 and 3DBK3-52) and introduced into these strains the *ARS CEN* plasmids bearing the RM and BY derived *MKT1* alleles. As shown in Figure 6, these strains displayed the same phenotype as was observed for the plasmid suppression in the parental third backcross strains, indicating that the *MKT1-BY* allele contributed to 4-NQO-sensitivity. We attempted to test suppression of the 4-NQO-sensitive phenotype in the backcrossed strains using homologous replacement of *MKT1;* however, we were unable to do so because the selectable marker used for integration interfered with *MKT1* function.

**Figure 7:**
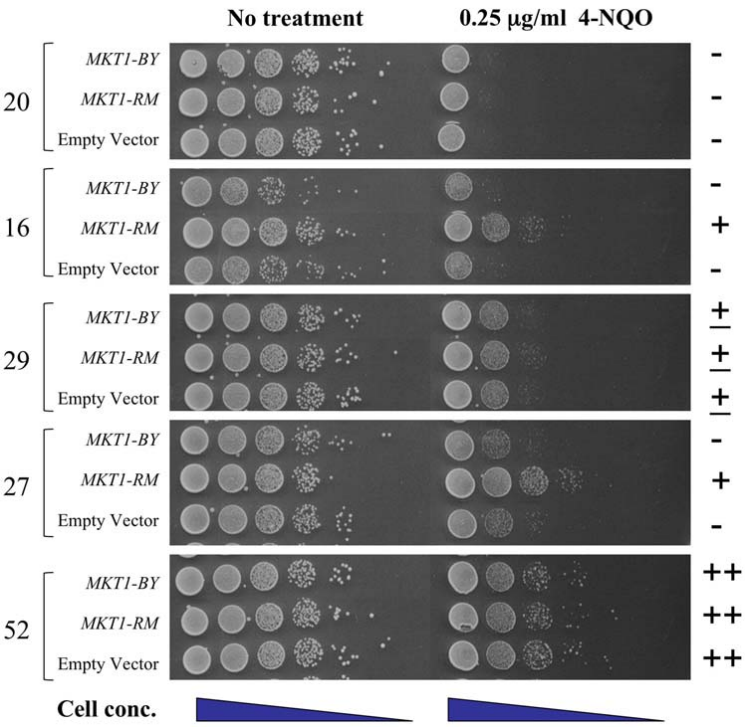
Allele-specific *MKT1* suppression of the 4-NQO-sensitive phenotype was observed in a fraction of BY/RM F1 hybrid progeny. Single-copy *ARS CEN* vectors containing BY or RM derived *MKT1* were introduced into F1 hybrid strains showing a range of 4-NQO phenotypes. Saturated cultures were diluted in water and spotted in 10-fold serial dilution (undiluted to 10^−5^) onto minimal selective yeast media with or without 0.25 µg/ml 4-NQO. Plates were photographed after 2-day incubation at 30 C.

**Figure 8:**
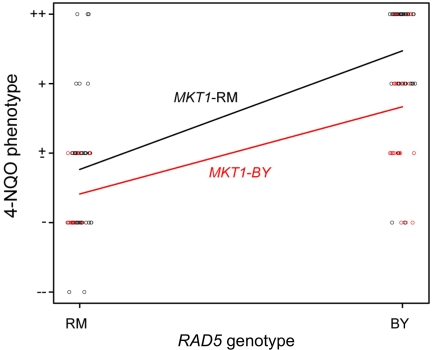
4-NQO sensitivity in meiotic spore progeny grouped by *RAD5* and *MKT1* genotype. The 4-NQO phenotypes of 123 spore progeny are plotted with respect to *RAD5*- and *MKT1*-region genotypes (Materials and Methods). This analysis shows that the loci do not strongly depend on each other.

Because we did not see a significant LOD score for the *MKT1* locus in the original linkage analysis, we suspected that additional alleles are contributing to sensitivity to 4-NQO in the original BY/RM hybrid progeny. To test this, we looked for allele-specific suppression by *MKT1-RM* in the original BY/RM meiotic spore progeny that showed sensitivity to 4-NQO (Figure 7). Some of the original hybrid progeny showed allele-specific suppression of 4-NQO-sensitivity (segregants 16 and 27, genotype *MKT1-BY* and *RAD5-BY*) while others did not (segregants 20 and 29, genotype *MKT1-RM* and *RAD5-RM*). In a resistant segregant, no phenotypic difference was seen between the plasmids (segregant 52, genotype *MKT1-RM* and *RAD5-BY*). These observations are consistent with multiple genes contributing to the 4-NQO sensitivity phenotype. Finally, to determine whether the *MKT1* and *RAD5* alleles show non-additive interaction with respect to sensitivity to 4-NQO, we plotted the semi-quantitative phenotype for 4-NQO shown in Table 1 against the *RAD5*- and *MKT1*-region genotypes (Figure 8). This analysis shows that the loci do not strongly interact (see Discussion) and that the most extreme genotypes are recombinant: the *RAD5*-RM, *MKT1*-BY genotype is the most sensitive, while the *RAD5*-BY, *MKT1*-RM genotype is the most resistant. This is consistent with what we observed in the backcrosses where the *MKT1*-BY allele was associated with sensitivity to 4-NQO in a *RAD5*-RM background (Figure 6).

## Discussion

We identified and dissected a complex trait, sensitivity to the DNA damaging agent 4-NQO, in hybrid progeny of baker's yeast. Through linkage mapping of genotyped hybrid progeny, we identified a major effect locus, *RAD5,* and identified the causative polymorphism. Using a backcrossing strategy along with microarray-assisted bulk segregant analysis we identified other QTL that contribute to the variability seen in the 4-NQO-sensitivity phenotype. The identification of a major effect locus likely contributed to our ability to map minor effect loci for sensitivity to 4-NQO.

Determining the number of loci that underlie a quantitative trait can be difficult due to epistasis, penetrance, and environmental factors that influence the phenotype. The 4-NQO- sensitive phenotype was seen at varying levels in about a quarter of the hybrid RM/BY progeny tested, indicating a minimum of two contributing loci. Backcrossing and bulk segregant analysis indicated that at least two other loci, including *MKT1*, contribute to a lesser degree to the sensitive phenotype observed in the hybrid progeny. The number of modifier QTL is likely greater than two due to the lack of suppression observed in some of the F1 hybrid progeny by *MKT1* (Figure 7).

After backcrossing, we were able to identify a large QTL region (∼118 kb) located on chromosome 7; however, a candidate gene approach was unsuccessful. There are many difficulties in finding functional variants in large QTL. Steinmetz et al. [8] identified two QTL in yeast (chromosome XIV, 52 kb, and chromosome XVI, 8 kb-not pursued further) that mapped to a heat resistance trait. Using reciprocal hemizygosity, they identified three genes in the chromosome XIV QTL linked to this phenotype. Using a backcrossing approach, Deutschbauer and Davis [38] identified four QTL in yeast (30-71 kb) linked to sporulation efficiency. They also performed reciprocal hemizygosity analysis and identified three genes linked to the phenotype. Finally, Perlstein et al. [10] mapped cell growth in the presence of 83 small molecule drugs to 219 QTL (42 kb on average) that localized to eight main regions in yeast. In their analysis they were able to identify two loci, primarily by candidate gene testing, that were linked to a particular drug sensitivity. Each of the above examples illustrates the complexity of mapping QTL to individual genes and the need for candidate gene testing. In our studies the large size (∼118 kb) of the Chromosome VII QTL suggests that candidate gene testing is likely to be very time consuming and more importantly, that the QTL is likely to be complex. The extended nature of the locus, despite extensive backcrossing, points to the possibility of a cluster of closely linked polymorphisms within the region causing the phenotype, making the identification of the individual genes difficult.

We found a major effect QTL in *RAD5,* and a minor effect modifier locus, *MKT1*. The *RAD5* gene was a logical candidate gene within its QTL because it is required for resistance to a number of DNA damaging agents and is a component of an extensively studied DNA repair pathway [25],[28],[29]. In contrast, *MKT1* has not been associated with any DNA damage pathway but has been identified in multiple QTL mapping studies performed in yeast, including sporulation and high temperature growth [3],[8],[37],[38]. In these studies, the same polymorphism in *MKT1* (D30G) was associated with the phenotype. Sinha et al. [3] hypothesized that BY derived *MKT1* is a loss of function allele and Deutschbauer et al. [38] showed that the *MKT1* polymorphism in the BY strain is rare; it was not found in 13 other *S. cerevisiae* strains. This same allele is likely to be involved in sensitivity to 4-NQO.

It is unclear how the *RAD5* and *MKT1* alleles interact to create the 4-NQO-sensitive phenotype in the hybrid progeny. The observations that these loci do not strongly interact each other, and that spore progeny displayed a wider range of sensitivity phenotypes, suggest an additive model for sensitivity to 4-NQO (Figure 8). In such a model, each parental strain contains negative and positive alleles, making them appear similar to each other. Segregation of such alleles in progeny would be expected to yield a wide range of phenotypes, as was seen. In this model, the 4-NQO-sensitive effect is transgressive rather than reflecting a defective interaction between the *MKT1* and *RAD5* gene products. Consistent with the above argument is the fact that genome-wide analyses of the response to DNA damaging agents (deletion, global expression studies) have not shown a direct connection between *RAD5* and *MKT1* that could explain the 4-NQO-sensitive phenotype with respect to known DNA repair pathways [40]–[42].

It is important to note that many QTL interactions cannot be easily explained in terms of an established genetic pathway. For example, for high temperature growth, the genes *MKT1, END3* (involved in endocytosis) and *RHO2* (a non essential GTPase) have been identified as QTL; how these three genes interact to confer heat resistance is unclear because no other genetic interactions involving these genes have been identified [3],[8]. *MKT1* has been shown to contribute to 4-NQO sensitivity, high temperature growth, and sporulation phenotypes. The fact that a single modifier could be involved in such a variety of phenotypes suggests that a candidate gene approach that tests previously identified modifiers should be considered when searching for loci that underlie a complex trait.

The *RAD5*-*RM* polymorphism conferred varying degrees of sensitivity to 4-NQO in RM/BY hybrid progeny. The scientific literature contains numerous examples in which an allele confers a more severe phenotype in one genetic background relative to another (e.g. [9],[14],[43]). In addition, environmental and background effects have been shown to affect the penetrance of many cancers (e.g. [11],[12]). Such effects are thought to be due to DNA sequence differences at multiple genetic loci that lead to molecular incompatibilities between gene products, between gene products and *cis*-acting sequences that function in specific pathways, negative epistatic interactions uncovered by haploinsufficiency, or as shown here, additive effects involving multiple QTL. Identifying such complex traits in a genetically tractable system is of great interest because they provide testable models to study disease penetrance (e.g. [3],[8],[17]).

## Materials and Methods

### Strains and Plasmids

*S. cerevisiae* parental strains, BY and RM, and BY/RM hybrid segregants (Table 1) were tested in this study [9]. Additional strains used in this study are listed in Table 2. Yeast strains were grown in yeast extract/peptone/dextrose (YPD), minimal complete or minimal selective media [44]. When required, nourseothricin (Werner Bioagents) or G418 sulfate (Cellgro) were included in YPD at 200 mg/l [44],[45]. Sporulation plates and procedures were as described previously [43],[46]. EAY253 (*rad52Δ::LEU2, ura3-52, leu2Δ1, his3Δ200*) was used as a control strain in the 4-NQO, MMS, bleomycin, and caffeine plating assays.

Plasmids used in this study are shown in Table 3. All of the plasmids are derived from the pRS vector series [47]. pRS317 was modified to contain the Gateway cloning cassette (Invitrogen). Each gene was amplified from BY or RM genomic DNA [44] using Pfu turbo polymerase (Stratagene). Primer sequences used to amplify these genes are available upon request. Each PCR product, which contained the entire open reading frame and at least 250 bp of upstream and downstream sequence, was gel purified and cloned into pENTR/D-TOPO entry vector (Invitrogen). The structure and sequence of all entry clones were verified by restriction endonuclease digestion followed by DNA sequencing. Subcloning of each gene from the pENTR/D-TOPO vector into pEAA403 (*LYS2, ARSH4, CEN6, aatR1::Cm^R^-ccdB::attR2)* was performed via LR recombination (Invitrogen). All of the resulting constructs were expressed via their native promoters. Plasmids were transformed into each strain using standard methods [48] and were selected for on lysine minimal dropout plates.

The *RAD5* integration vectors, pEAI209 (*RAD5*-*BY*::*NatMX*) and pEAI210 (*RAD5*-*RM*::*NatMX*) contain *RAD5* amplified from BY and RM genomic DNA, respectively [44]. The sequences were amplified using Pfu turbo polymerase (Stratagene) and primers AO990 (5′CAGGACACTGACAACGAATTGC) and AO991 (5′GTTTGCGTTAGAGCAATTCC). The PCR amplified product containing the entire *RAD5* open reading frame plus 450 bp upstream and 700 bp downstream sequence was digested with *PstI* and *SalI* and inserted into the corresponding sites of pUC19. The entire PCR fragment was DNA sequenced. Using overlapping PCR and subcloning, *BamHI* and *NotI* sites were added 30 bp downstream of the *RAD5* stop codon using AO946 (5′GAGAAAGAGCTAACTCATACTT), AO1023 (5′CGACTAGTGCGGCCGCTAGTCGGGATCCAAAGTCTTTATATATGAGTATG), AO1024 (5′CGACTAGTGGATCCCGACTAGCGGCCGCATTTATTATTATTTTCAACC) and AO616 (5′CGCCATTCAGGCTGCGCAACT). The *NatMX* gene from pAG25 [49] was then inserted into these *Bam*H1 and *Not*I sites. Integration plasmids were also made that contained *KanMX* downstream of *RAD5*, pEAI207 (*RAD5*-BY::*KanMX*) and pEAI208 (*RAD5*-RM::*KanMX*), using the same procedure. All *RAD5* point mutations were made in pEAI207 using the QuickChange XL Site-Directed Mutagenesis protocol (Stratagene, USA). A fragment containing the point mutation was then subcloned into unmutagenized pEAI207 and sequenced to determine that only the desired mutation was created.

For homologous replacement of *RAD5*, integration plasmids pEAI209, pEAI210, pEAI213, and pEAI214 were digested with *XbaI* and *NheI* and the fragments were transformed into BY and RM using standard methods [48]. Integrations were selected on YPD media containing G418 sulfate or nourseothricin [45]. Each allele was PCR amplified and sequenced to determine that only the desired mutations were created.

Two strains from the third backcross of RM×BY-*RAD5-RM*, 3DBK3-12 (EAY2295) and 3DBK3-52 (EAY2298), were transformed with a *mkt1Δ::NatMX* DNA fragment to create EAY2317 and EAY2323, respectively (Table 2). This fragment contains *NatMX* flanked by 50 bp of *MKT1* sequence upstream of the *MKT1* ATG and 50 bp of *MKT1* sequence downstream of the *MKT1* stop codon. It was created by PCR amplifying pAG25 with primers AO2014 (5′TGAACTATAAAGTACTAAAGGCAGAAAAATTAATAGCAAATTAAGCGATGCGTACGCTGCAGGTCGAC) and AO2015 (5′TGCTTTTTAAATAGTTCCACTATTTCCATCATA CTCATTCTCACGCTTCAA TCGATGAATTCGAGCTCG). Integrations were selected for on YPD media containing nourseothricin and the *mkt1Δ::NatMX* mutation was confirmed by PCR.

### Plating Assays

RM, BY, and hybrid segregants were grown to saturation in YPD liquid media. The cultures were then diluted in water and spotted in 10-fold serial dilutions (undiluted to 10^−5^) onto YPD media containing 0.03% MMS (vol/vol) (Sigma), 0.25 µg/ml or 0.30 µg/ml 4-NQO (Sigma), 10 mM caffeine (Sigma), or 10 µg/ml bleomycin (Sigma) [22]. Plates were photographed after a 2-day incubation at 30 C. Each segregant was scored according to growth on non-treatment and treatment plates and were tested at least three independent times for each phenotype reported in Table 1. Phenotypes were scored for linkage analysis using a semi-quantitative method based on the phenotypes reported in Table 1 in which each phenotype group was assigned a unique category (below).

### Linkage Analysis

Linkage analysis on 121 segregants with genotype information was performed in R/qtl [50] using the non-parametric model. Genotypes were previously generated by DNA hybridization to Affymetrix 25-mer arrays [20]. Permutations (4000x per phenotype) were performed to obtain a significance cutoff where p = 0.0125 (0.05/the number of traits: 4). This corresponded to LOD scores of 3.9, 3.8, 3.8, and 3.7 for MMS-, 4-NQO-, bleomycin-, and caffeine-sensitivity, respectively (Figure 2). Support intervals defined by a 1.5 drop in LOD score from the peak were calculated using the lodint function in R/qtl.

### Tiling Array Analysis

DNA samples were prepared by pooling 5 ml YPD overnight cultures from 40 backcrossed individuals that were resistant and 40 that were sensitive to 4-NQO. Genomic DNA was prepared separately from the resistant and sensitive pooled cultures using the QIAGEN Genomic-tip 500/G kit (Qiagen). The pooled gDNA samples were hybridized to Affymetrix tiling arrays containing Watson strand 25-mers tiled every 4 bp (BY reference genome). The log_10_ ratio of intensity values was calculated for every probe that was considered to contain a sequence variant between BY and RM by SNPscanner [51]. The log_10_ (intensity difference) was plotted along the chromosome and smoothed using the smooth.spline function in the stats package of R. Regions where the smoothed value exceeded +/−1 were further investigated.

## Supporting Information

Figure S1 Homologous Replacement of *RAD5* in BY and RM strain backgrounds. Saturated cultures of the indicated strains were diluted in water and spotted in 10-fold serial dilution (undiluted to 10^−5^) onto YPD media with or without 0.03% MMS. The plates were photographed after a 2-day incubation at 30 C.
(1.07 MB TIF)

Figure S2 Plasmid Replacement of *RAD5* in RM strain background. Saturated cultures of RM, BY and RM strains containing *RAD5-RM* or *RAD5-BY* on *ARS CEN* plasmids were diluted in water and spotted in 10-fold serial dilution (undiluted to 10^−5^) onto YPD media with or without 0.05% MMS. Plates were photographed after a 2-day incubation at 30 C.
(0.70 MB TIF)

Figure S3 Homologous Replacement of *RAD5* in BY and RM strain backgrounds. Saturated cultures of the indicated genotypes were diluted in water and spotted in 10-fold serial dilution (undiluted to 10^−5^) onto YPD media with or without 0.05% MMS. The plates were photographed after a 2-day incubation at 30 C.
(1.02 MB TIF)

Figure S4 No candidate genes showed suppression of 4-NQO-sensitivity. The BY::*RAD5-RM* strain transformed with candidate genes present on ARS CEN vectors were diluted in water and spotted in 10-fold serial dilution (undiluted to 10^−5^) onto selective media with or without 0.25 µg/ml 4-NQO. Plates were photographed after a 2-day incubation at 30 C.
(1.03 MB TIF)
