# Supplementary figures and images for: Identification and Dissection of a Complex DNA Repair Sensitivity Phenotype in Baker's Yeast
Source: PLoS Genet. 2008 Jul 11;4(7):e1000123. doi: 10.1371/journal.pgen.1000123 (PMC2440805; doi:10.1371/journal.pgen.1000123)

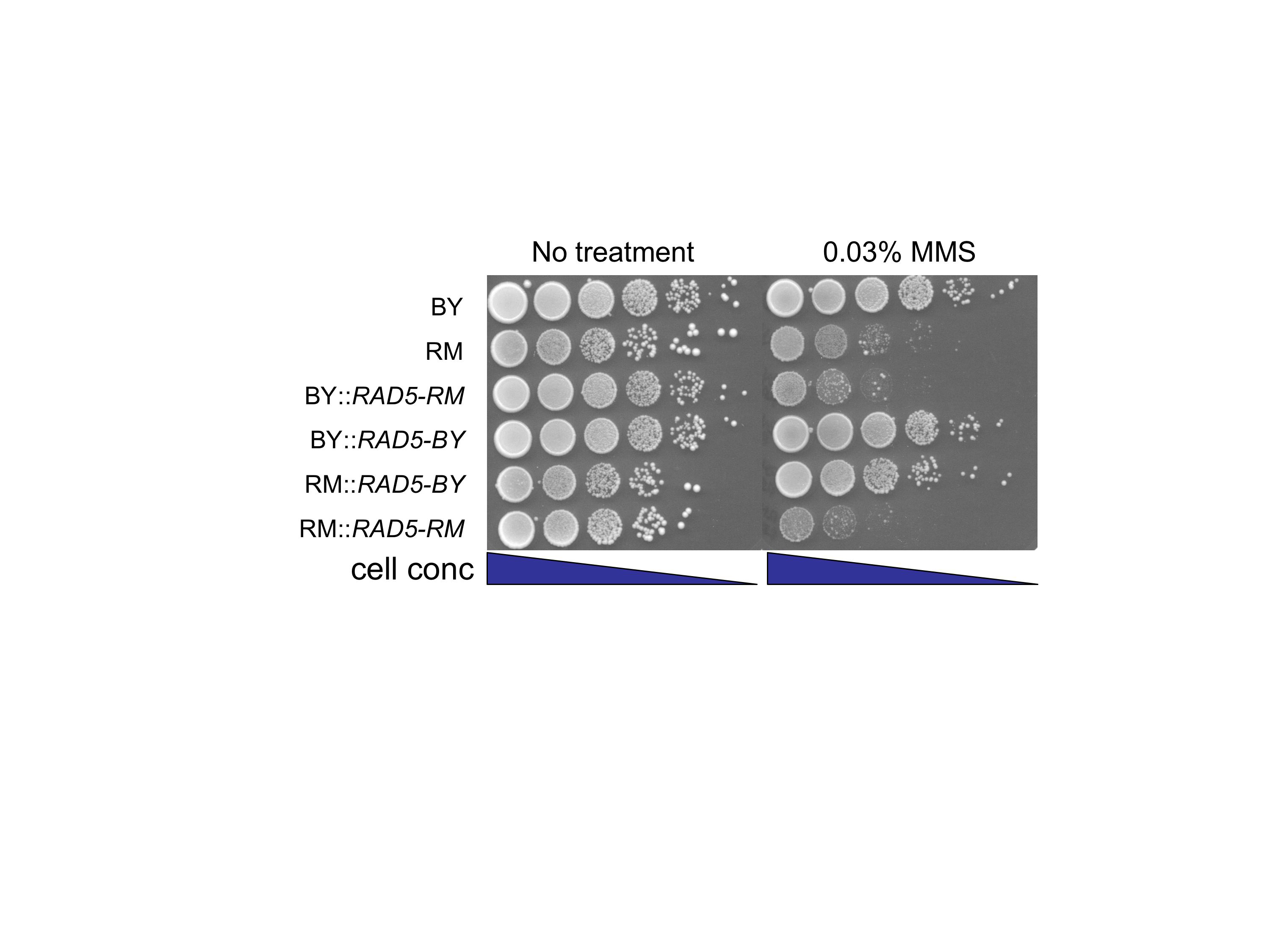

Supplement: Figure S1 — Homologous Replacement of RAD5 in BY and RM strain backgrounds. Saturated cultures of the indicated strains were diluted in water and spotted in 10-fold serial dilution (undiluted to 10−5) onto YPD media with or without 0.03% MMS. The plates were photographed after a 2-day incubation at 30 C. (1.07 MB TIF) [file pgen.1000123.s001.tif]

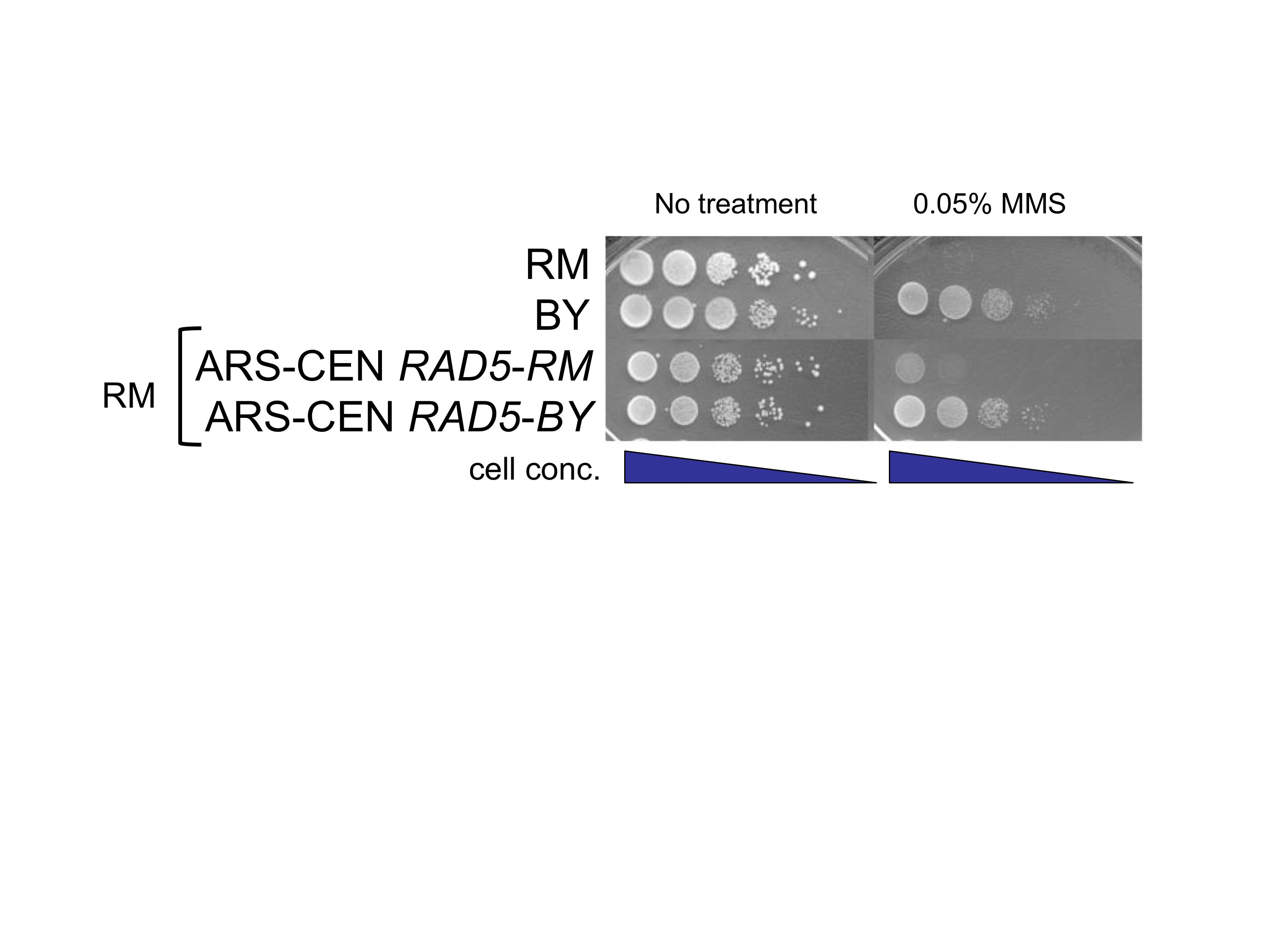

Supplement: Figure S2 — Plasmid Replacement of RAD5 in RM strain background. Saturated cultures of RM, BY and RM strains containing RAD5-RM or RAD5-BY on ARS CEN plasmids were diluted in water and spotted in 10-fold serial dilution (undiluted to 10−5) onto YPD media with or without 0.05% MMS. Plates were photographed after a 2-day incubation at 30 C. (0.70 MB TIF) [file pgen.1000123.s002.tif]

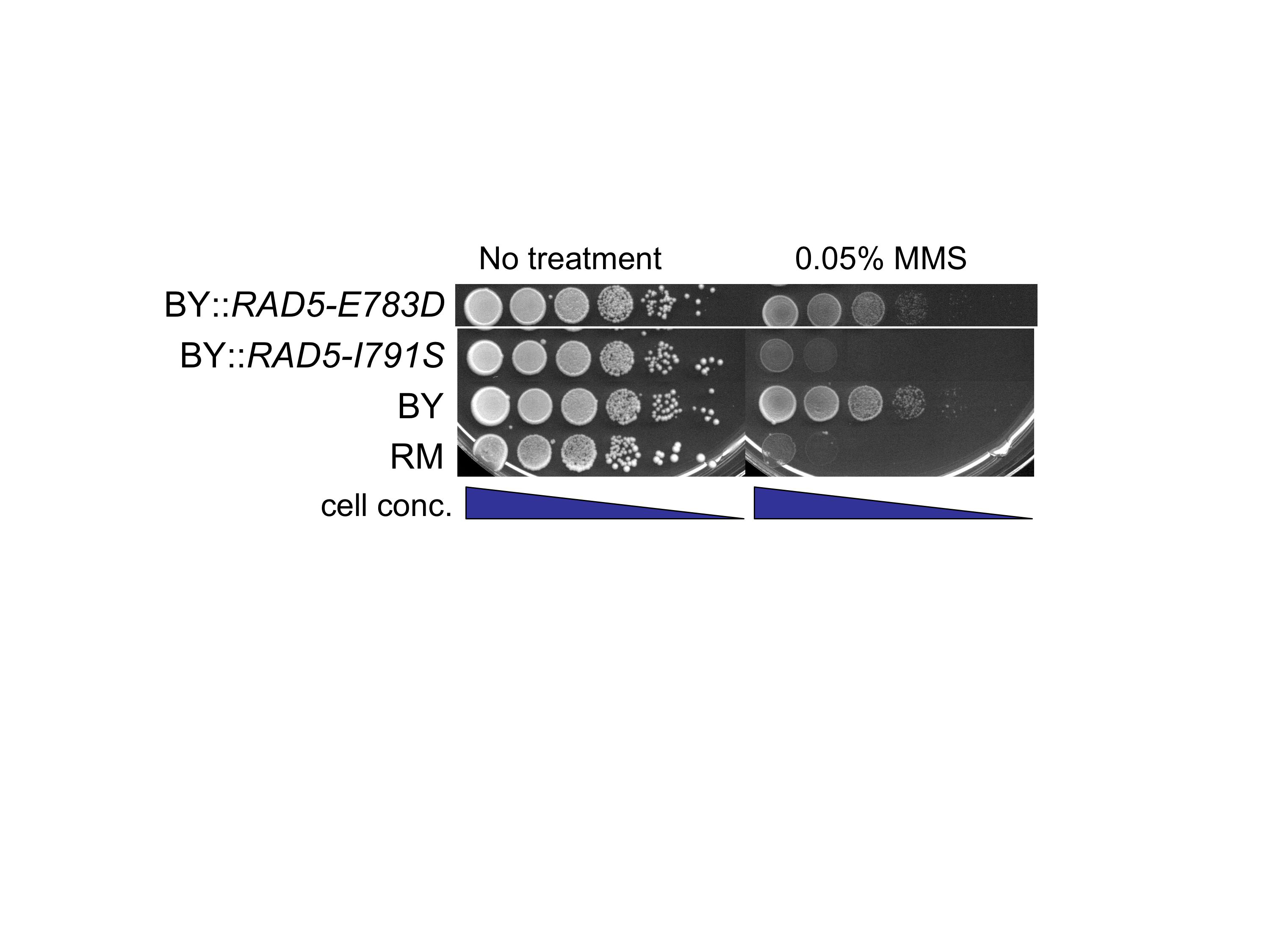

Supplement: Figure S3 — Homologous Replacement of RAD5 in BY and RM strain backgrounds. Saturated cultures of the indicated genotypes were diluted in water and spotted in 10-fold serial dilution (undiluted to 10−5) onto YPD media with or without 0.05% MMS. The plates were photographed after a 2-day incubation at 30 C. (1.02 MB TIF) [file pgen.1000123.s003.tif]

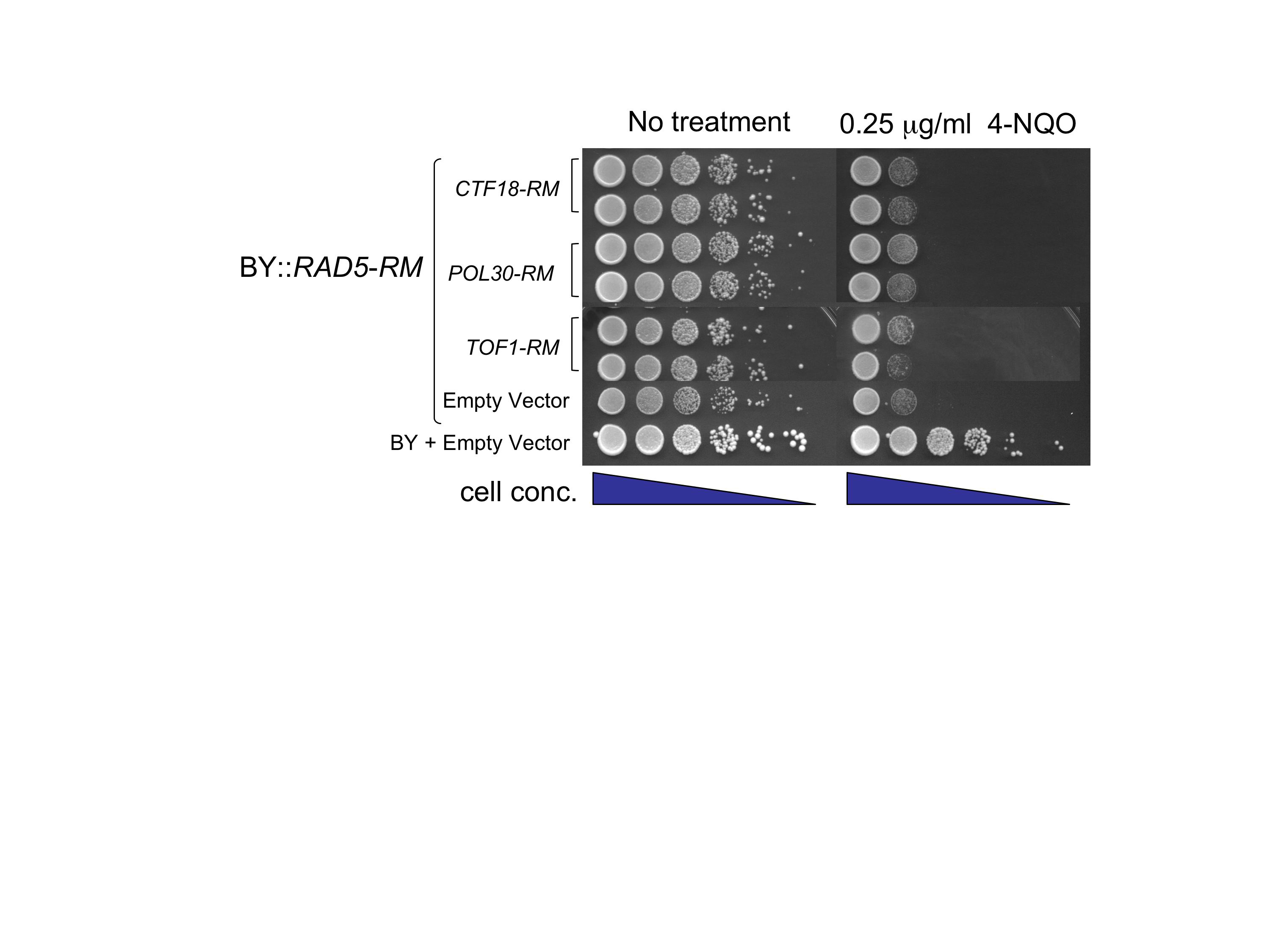

Supplement: Figure S4 — No candidate genes showed suppression of 4-NQO-sensitivity. The BY::RAD5-RM strain transformed with candidate genes present on ARS CEN vectors were diluted in water and spotted in 10-fold serial dilution (undiluted to 10−5) onto selective media with or without 0.25 µg/ml 4-NQO. Plates were photographed after a 2-day incubation at 30 C. (1.03 MB TIF) [file pgen.1000123.s004.tif]
